# Early Mortality of Prostatectomy vs. Radiotherapy as a Primary Treatment for Prostate Cancer: A Population-Based Study From the United States and East Germany

**DOI:** 10.3389/fonc.2019.01451

**Published:** 2020-01-17

**Authors:** Daniel Medenwald, Dirk Vordermark, Christian T. Dietzel

**Affiliations:** Department of Radiation Oncology, University Hospital Halle (Saale), Halle (Saale), Germany

**Keywords:** early mortality, prostate cancer, prostatectomy, radiotherapy, general population

## Abstract

**Objective:** To assess the extent of early mortality and its temporal course after prostatectomy and radiotherapy in the general population.

**Methods:** Data from the Surveillance, Epidemiology, and End Results (SEER) database and East German epidemiologic cancer registries were used for the years 2005–2013. Metastasized cases were excluded. Analyzing overall mortality, year-specific Cox regression models were used after adjusting for age (including age squared), risk stage, and grading. To estimate temporal hazards, we computed year-specific conditional hazards for surgery and radiotherapy after propensity-score matching and applied piecewise proportional hazard models.

**Results:** In German and US populations, we observed higher initial 3-month mortality odds for prostatectomy (USA: 9.4, 95% CI: 7.8–11.2; Germany: 9.1, 95% CI: 5.1–16.2) approaching the null effect value not before 24-months (estimated annual mean 36-months in US data) after diagnosis. During the observational period, we observed a constant hazard ratio for the 24-month mortality in the US population (2005: 1.7, 95% CI: 1.5–1.9; 2013: 1.9, 95% CI: 1.6–2.2) comparing surgery and radiotherapy. The same was true in the German cohort (2005: 1.4, 95% CI: 0.9–2.1; 2013: 3.3, 95% CI: 2.2–5.1). Considering low-risk cases, the adverse surgery effect appeared stronger.

**Conclusion:** There is strong evidence from two independent populations of a considerably higher early to midterm mortality after prostatectomy compared to radiotherapy extending the time of early mortality considered by previous studies up to 36-months.

## Introduction

Prostate cancer is by far the most common malignancy in males, with 161,360 new cases diagnosed annually in the United States (US) (1). Though early detection of the disease has improved inter alia due to screening programs, prostate cancer still contributes to about 8% of all cancer deaths in US males, which is only surpassed by colon and lung tumors.

Primary treatment of low-risk disease patients may include surgery, external radiotherapy, low-dose brachytherapy, and, in certain cases, active surveillance.

While prostatectomy seems to be the more frequently utilized treatment in Germany, patients in the US receive both radiotherapy and surgery in equal proportions (2). Comparing the US Surveillance, Epidemiology, and End Results (SEER) data with German cancer registries, Hager et al. (2) estimated the proportion of patients receiving prostatectomy as 36.1 vs. 66.2% and radiotherapy as 38.4 vs. 11.8%, respectively, of identified cases.

Nevertheless, there is insufficient evidence from randomized controlled trials comparing the results of the different therapeutic options. Both the Scandinavian SPCG-4 and the US PIVOT-trial failed to show a cancer-specific survival benefit in low-risk disease if a surgical treatment was set against an observational concept of watchful waiting (3, 4). A German benchmark study called “Prefere,” which aimed to compare all four primary types of therapy, had to be closed due to a lack of recruitment (5). It was not until recently that the British “ProtecT” trial finally confirmed comparable results regarding all-cause mortality following surgery, external radiotherapy, and active surveillance (6). However, it is important to mention that the adverse effects differed strikingly (7).

All studies were reporting long-term outcomes, while it seems reasonable that also short-term results are of important interest, as they could guide treatment decisions especially for elderly or multi-morbid patients.

We hypothesize that there is an increased early mortality following primary prostatectomy reflecting differing periprocedural risks compared to radiotherapy or observation in daily clinical practice. This is especially important as an early mortality might be distinct from long-term mortality in terms of both treatment-related mortality and mortality pattern where procedure-associated deaths might dominate in an early phase.

Thus, it is the aim of this study to analyze data from two national cancer registries, as they can offer unbiased population data on treatment and survival (8). In contrast to the strict inclusion criteria of randomized controlled trials, they comprise a broad range of patients in respect to age, comorbidities, and the catchment area of medical services, which limits a possible referral bias.

In this study, we focused on the SEER database to analyze this effect. In order to verify the findings in an independent cohort, we considered data from German cancer registries. Effect estimates of comparable size in both cohorts would support the validity of results and limit biases due to cohort heterogeneity. This supports evidence that results are not subject to biases related to contextual public health factors.

## Methods

### Data

We used data from the SEER registry covering prostate cancer cases in the USA and epidemiologic cancer data from the Robert Koch Institute in Germany for the federal states of East Germany. The observational period encompassed the years from 2005 to 2013.

### Inclusion Criteria

Cases with metastases at time of diagnosis, with a cancer diagnosis based solely on a death certificate or autopsy, or cases that received chemotherapy as the initial treatment were excluded from the analysis. In addition, subjects that received both surgery and radiotherapy (such as an adjuvant treatment) were excluded from the analyses. In the German cohort, data from East Germany, including Berlin, were used. These registries distinguish themselves by high coverage of incident cases and data quality (2).

Finally, 440,987 cases from the SEER database and 71,020 cases from East German registries fulfilled these criteria. Of these, 411,456 cases (93.3%) from the SEER database and 58,161 cases (81.9%) from the German registries had no missing values.

### Variable Definition

In our analyses, “treatment” refers to the initial treatment. We chose the time of diagnosis as the start of follow-up in order to avoid an immortal time bias (time from diagnosis to initiation of treatment) (9). No treatment was defined when subjects received neither surgery nor radiotherapy, thus incorporating cases eligible for active surveillance or unfit for treatment because of a poor health condition.

Low risk was defined according to EAU Guidelines (10) and previous work: (2) stage T1/2a, N0/X, M0/X, and Gleason score ≤ 6 corresponding to Grade 1/2 (as coded in German cancer registries, see for the SEER data: https://seer.cancer.gov/tools/grade/). We used the derived AJCC, 6th edition (2004+) stage data to determine stages (11).

### Statistical Analyses

Long-term survival of patients diagnosed in 2005 was compared between surgery and radiotherapy by propensity score matching. We used a logistic regression model to compute propensity scores as the estimated probability of receiving radiotherapy. In this model, we accounted for the following factors: age, age squared, grading differentiation between a Gleason score ≤ 6 and above, and T-stage, respectively, allowing for the interaction between year of diagnosis and each main effect covariate (separate models for each year of diagnosis, relevant parameters identified by means of backward selection). For the matching procedure, we used the SAS macro by Parsons et al. (12), which uses the following criterion: The algorithm matches cases iteratively to their best possible match (controls) based on an eight-digit propensity score in a hierarchical manner. That is, if no match is found in the first round, the second best match is used and so forth (without reconsidering already matched pairs) (12). After matching, the cohort consisted of 64,156 cases per group in the SEER cohort and 7,950 cases per group in the German data.

**Figure 1:**
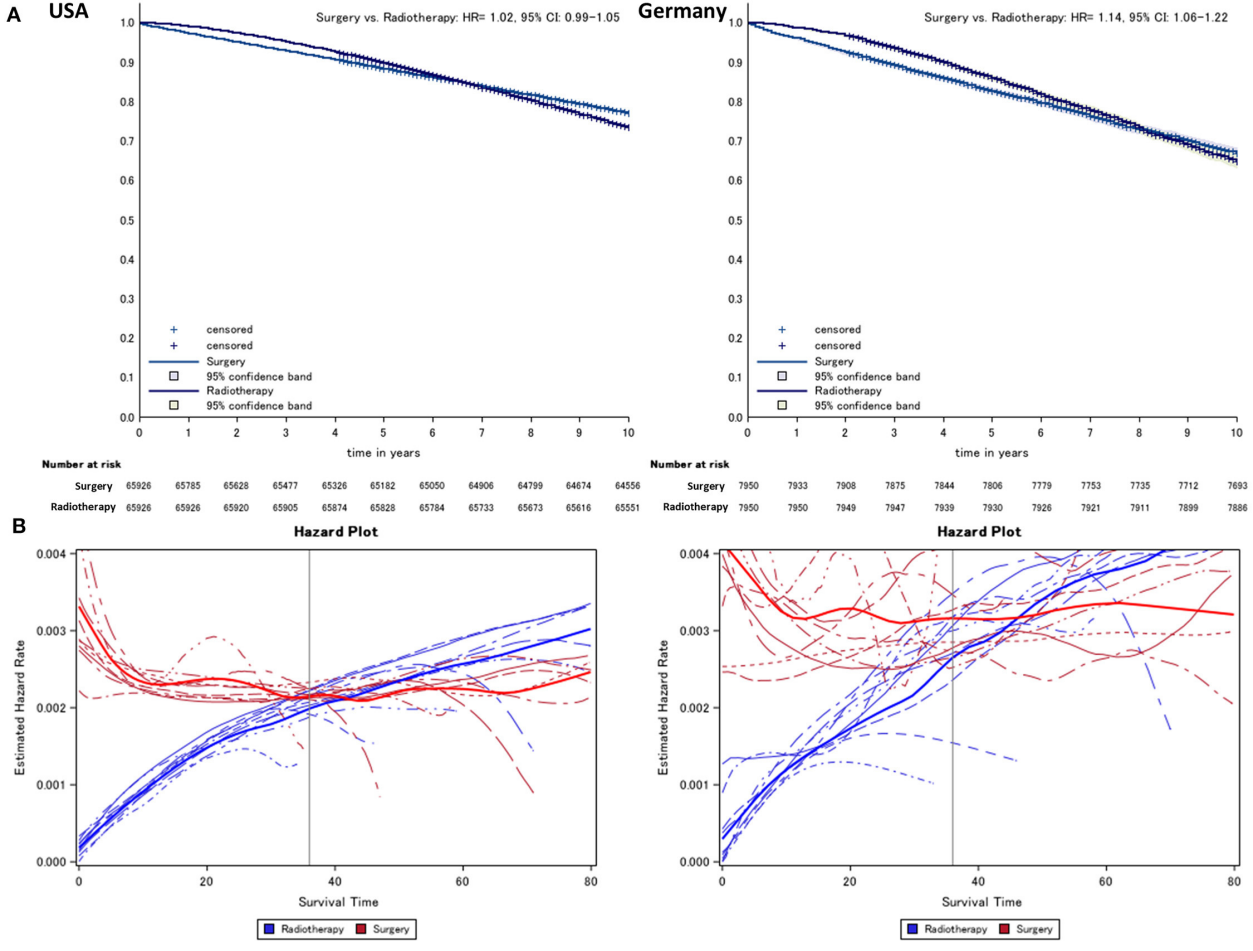
Kaplan–Meier **(A)** comparing surgery vs. radiotherapy (radiotherapy = reference) and **(B)** hazard survival plot of prostate cancer patients from German and US cancer registries. Lines indicate the year of diagnosis specific (2005–2013) conditional hazard for radiotherapy (risk to die in the following month, blue) and surgery (red) from Kaplan–Meier plots. Thick lines refer to the smoothed average of year-specific hazard in patients treated with radiotherapy and surgery, respectively.

After matching, in survival curves of both regions, an early phase where radiotherapy is superior to surgery can be distinguished from a later period where relations are reversed (Figure 1). To determine the end of this phase, the year-specific hazards are displayed, distinguishing between surgery and radiotherapy (Figure 1). Here, we found the time point at which hazards start to run in parallel to be around 24-months after diagnosis at the earliest, while the estimated annual mean was around 36-months (check for each imputation, see below). Thus, we define the time before 24-months after diagnosis as early mortality (24-month mortality is referenced by the term “early mortality” throughout the manuscript; otherwise, the observational times are mentioned explicitly).

Absolute risk differences (RDs) were obtained from Kaplan–Meier estimates after propensity score matching as the difference between estimated survival probabilities at distinct time points (3, 6, and 12-months, and every 12-months subsequently).

### Piecewise Proportional Hazard Models and Treatment Effect in Relation to Time After Diagnosis

To analyze the temporal course of hazards, we applied piecewise proportional hazard models by considering periods of 3, 6, 12, 24, and 36-months in the outcome time variable of a Poisson regression model with time since diagnosis as an offset.

### Early Mortality and Year of Diagnosis (2005–2013)

For year-specific survival analyses, we used Cox regression models estimating the average of the hazard ratio (HR) during the time of 24-months (earliest observed crossing of hazards curves) for the years of diagnosis between 2005 and 2013 according to the time of diagnosis. Models were adjusted for age, age squared, grading differentiation between a Gleason score below and above 6, and risk (low vs. intermediate and high), respectively, allowing for the interaction between year of diagnosis and each main effect covariate, and thus for a temporal variation of potential confounders.

### Multiple Imputation and Missing Data

We used multiple imputation (five imputations) to impute missing data taking the variables of T-stage and histopathologic grading, age, and treatment into account. Missing data were mostly confined to the grading parameter in the German data (17.73%, Table S1). For propensity score matched analyses (figures of individual data, that is, Kaplan–Meier curves and hazard plots, show complete case data) and year-specific Cox regression analyses, we used PROC MI in SAS. In piecewise proportional hazard models, we applied the “mice” package in R. Matching results for the complete data are shown in Table S2, which indicate equal mean values and proportions between treatment groups.

### Additional and Sensitivity Analyses

We performed an analysis of missing data in relation to treatment by using logistic regression models considering treatment, age, and year of diagnosis as covariates (Figure S1). As in few cases, prostate cancer might have been diagnosed secondarily after prostatectomy as part of the surgical treatment for bladder cancer; we performed a sensitivity analysis where cases that died from bladder cancer were excluded from the analysis. Furthermore, we performed a sensitivity analysis of cases with low-risk prostate cancer and an age below 60-years taking only US data into account as there were few deaths in this sub-cohort in the German data. Here, we computed relative risks as HRs from piecewise regression and absolute RDs (see above).

All statistical analyses were performed using R 3.1.2 (R Core Team 2015, Vienna, Austria) and SAS^®^, Version 9.3 (SAS Inc., Cary, NC, USA). We presumed a level of significance of 5% and thus report 95% confidence intervals (CI).

## Results

### Basic Characteristics (Table 1)

Regarding the SEER data, the proportion of patients that were alive 24-months after diagnosis was higher in the radiotherapy (97.2%) and surgery group (97.0%) than in the observation arm (92.5%). Results in Germany were comparable (radiotherapy: 96.7%, surgery: 96.1% vs. observation: 90.7%).

**Table 1:** Characteristics of the study population including the proportion of subjects alive 24-months after diagnosis and the proportion of causes of death among deceased patients according to the SEER causes of death coding.

	**No treatment**		**Radiotherapy**		**Surgery**	
	**Mean [95% CI]**		**Mean [95% CI]**		**Mean [95% CI]**	
**USA**						
Age	69.7 [69.7–69.8]		68.2 [68.2–68.3]		63.0 [63.0–63.1]	
**Frequency values**						
	***n***	**%**	***n***	**%**	***n***	**%**
Gleason ≤ 6	57,327	60.3	65,289	45.3	69,994	40.6
Gleason >6	37,737	39.7	78,818	54.7	102,291	59.4
Locally limited (T1/T2)	61,493	96.6	113,517	96.0	117,697	79.9
Locally advanced (T3/T4)	1,286	2.0	3,801	3.2	26,621	18.1
Node-positive	899	1.4	990	0.8	2,937	2.0
Alive after 24-months	87,930	92.5	140,120	97.2	167,099	97.0
**Causes of death**						
Prostate	1,931	22.2	1,061	25.5	1,025	18.1
Diseases of heart	1,731	19.9	526	12.6	819	14.4
Other cause of death	808	9.3	391	9.4	471	8.3
Lung and bronchus	95	1.1	22	0.5	1,043	18.4
Miscellaneous malignant cancer	569	6.5	344	8.3	241	4.3
**GERMANY**						
Age	72.7 [72.6–72.9]		71.3 [71.1–71.4]		66.8 [66.7–66.9]	
**Frequency values**						
Gleason ≤ 6	8,799	69.2	6,252	73.3	25,840	70.0
Gleason >6	3,915	30.8	2,270	26.6	11,076	30.0
Locally limited (T1/T2)	8,083	83.2	5,964	86.5	22,858	75.5
Locally advanced (T3/T4)	1,393	14.3	751	10.9	6,078	20.1
Node-positive	244	2.5	178	2.6	1,334	4.4
Alive after 24-months	11,536	90.7	8,282	97.2	35,508	96.2
**Causes of death**						
Unknown	532	45.1	130	53.5	709	50.2
Prostate	169	14.3	25	10.3	178	12.6
Symptoms, signs and ill-defined conditions	159	13.5	27	11.1	88	6.2
Diseases of heart	18	1.5	1	0.4	182	12.9
Lung and bronchus	46	3.9	6	2.5	31	2.2

Differences between treatment groups (Table 1) were mostly confined to deaths due to diseases of the heart and other causes and bladder cancer in cases that received surgery (see also the sensitivity analysis in Figure 5). In the German cohort, relations appeared to be less clear due to a high number of non-specified causes.

### Long-Term Survival and Time-Specific Analyses (Figures 1, 2)

Over the entire observational period, we found on average a lower overall mortality in the radiotherapy group compared to surgery after propensity score matching in both cohorts (USA: HR = 1.0, 95% CI: 0.99–1.05, Germany: HR = 1.14, 95% CI: 1.06–1.22, Figure 1).

**Figure 2:**
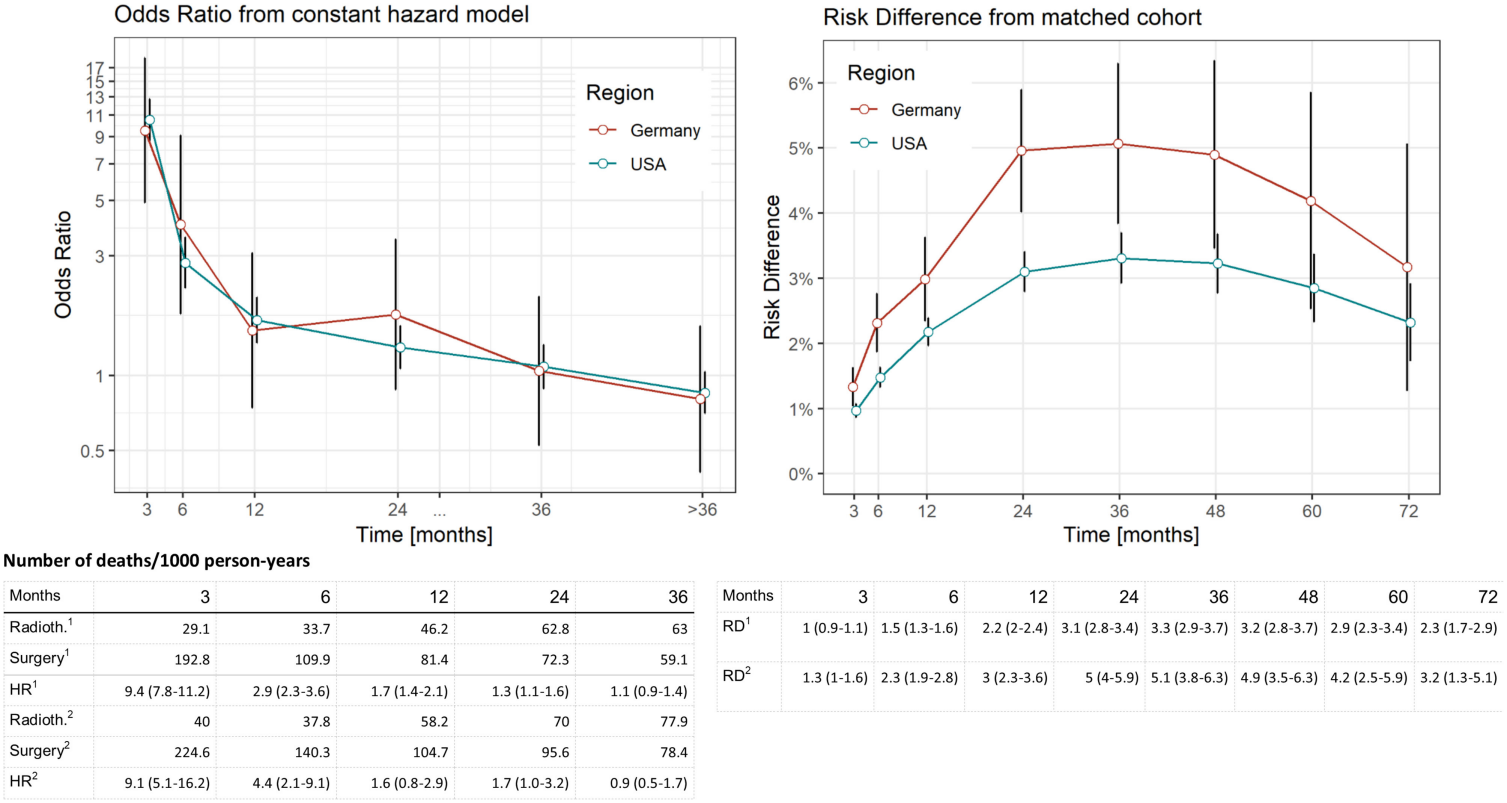
Comparison of distinct survival times of surgery vs. radiotherapy in the SEER data (USA) and German Registries. Estimates computed by means of piecewise proportional hazard models based on a Poisson regression. Vertical lines reflect 95% confidence intervals. Deaths per 1,000 person years in relation to region (1: USA, 2: Germany) and treatment. Risk difference was computed from Kaplan–Meier estimates after propensity score matching at respective time points (vertical lines reflect 95% confidence intervals).

In the piecewise proportional hazards model, we found an HR of 9.4 (95% CI: 7.8–11.2, Figure 2) to die within 3-months after diagnosis in the US data and an HR of 9.1 (95% CI: 5.1–16.2, Figure 2) in the German data. In the German cohort, the null effect value was not reached 20-months after diagnosis.

When RDs as an absolute risk estimate are concerned (Figure 2), we found an increase reaching a plateau of 24-months after diagnosis in both cohorts (RD ranging between 3.1 and 3.3 in the SEER and 4.9–5.1 in the German data for a period of 24–48-months after diagnosis). Comparing these findings to relative risk estimates, the beginning of the plateau phase corresponds to the last time point when mortality risks are higher in the surgery group (24-months after diagnosis, Figure 2), while the plateau itself reflects equal risks in subsequent months when the accumulation of deaths are similar in both treatment groups.

### Early Mortality and Year of Diagnosis (Figure 3)

**Figure 3:**
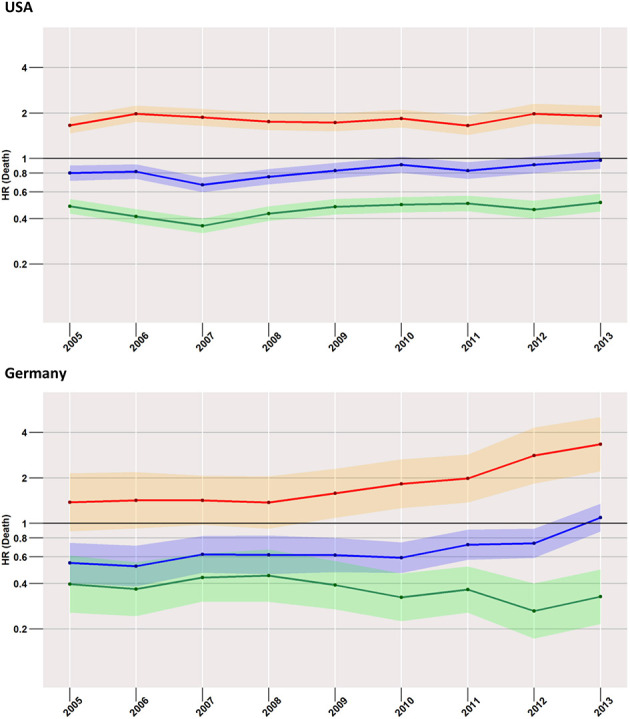
Treatment comparisons between radiotherapy, surgery, and no treatment for overall early mortality (24-months) of the years between 2005 and 2013. Adjusted models, Red, surgery vs. Radiotherapy; Blue, surgery vs. no treatment; Green, radiotherapy vs. no treatment.

Starting with the US data, during all considered years, we found a disadvantageous situation for patients treated with surgery when compared to radiotherapy (red curve in Figure 3), with an HR of 1.7 (95% CI: 1.5–1.9) computed for 2005, changing little to 1.9 (95% CI: 1.6–2.2) in 2013. However, the surgery group experienced a better survival rate when compared to the no-treatment group (blue curve). Better still was the average early survival estimate of the radiotherapy group when compared to the group of subjects having received no treatment.

This situation was mirrored in the German cohort, where surgery was again related to higher mortality when compared to radiotherapy, which was true for all years taken into account (2005: 1.4, 95% CI: 0.9–2.1; 2013: 3.3, 95% CI: 2.2–5.1).

### Low-Risk Cases (Figure 4)

Focusing on the low-risk group, the most striking difference to the analysis of the total cohort was found in US patients when surgery and no treatment were compared. Here, the risk of death within 24-months after diagnosis was higher in the surgery group than in the group with no treatment. The effect increased slightly over time from an HR of 1.1 (95% CI: 0.9–1.4) in 2005 to an HR of 1.7 (95% CI: 1.3–2.2) in 2013. In the German population, estimates undulated around an HR of 1, with 95% CIs covering it across all considered years.

**Figure 4:**
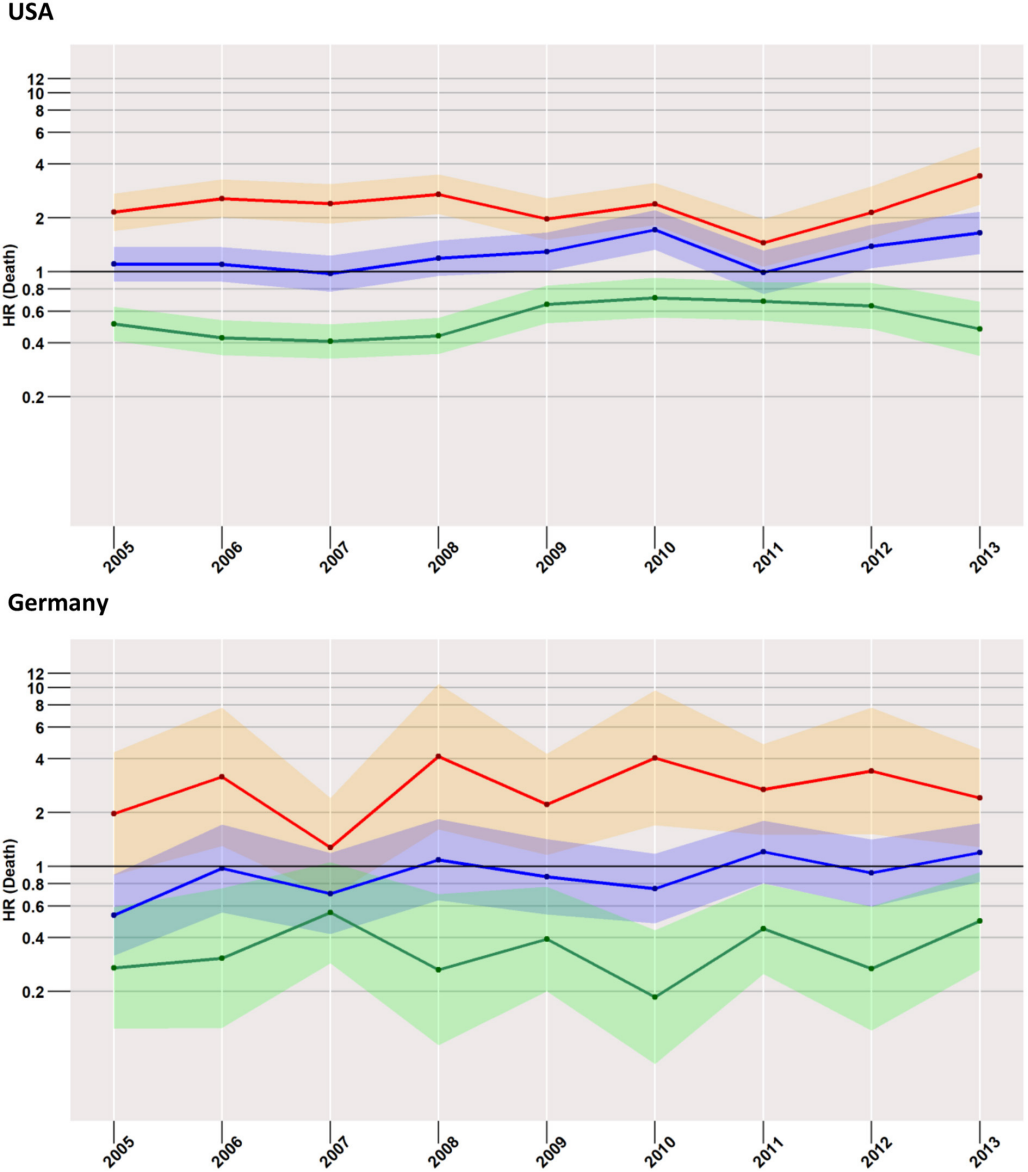
Treatment comparisons of low-risk patients between radiotherapy, surgery, and no treatment for overall early mortality (24-months) of the years between 2005 and 2013. Adjusted models, Red, surgery vs. radiotherapy; Blue, surgery vs. no treatment; Green, radiotherapy vs. no treatment.

Compared to radiotherapy, surgery had a considerably higher mortality rate (HR for SEER in 2013: 3.4, 95% CI: 2.4–5.0; German registries in 2013: 2.4, 95% CI: 1.3–4.5).

### Additional and Sensitivity Analyses

**Figure 5:**
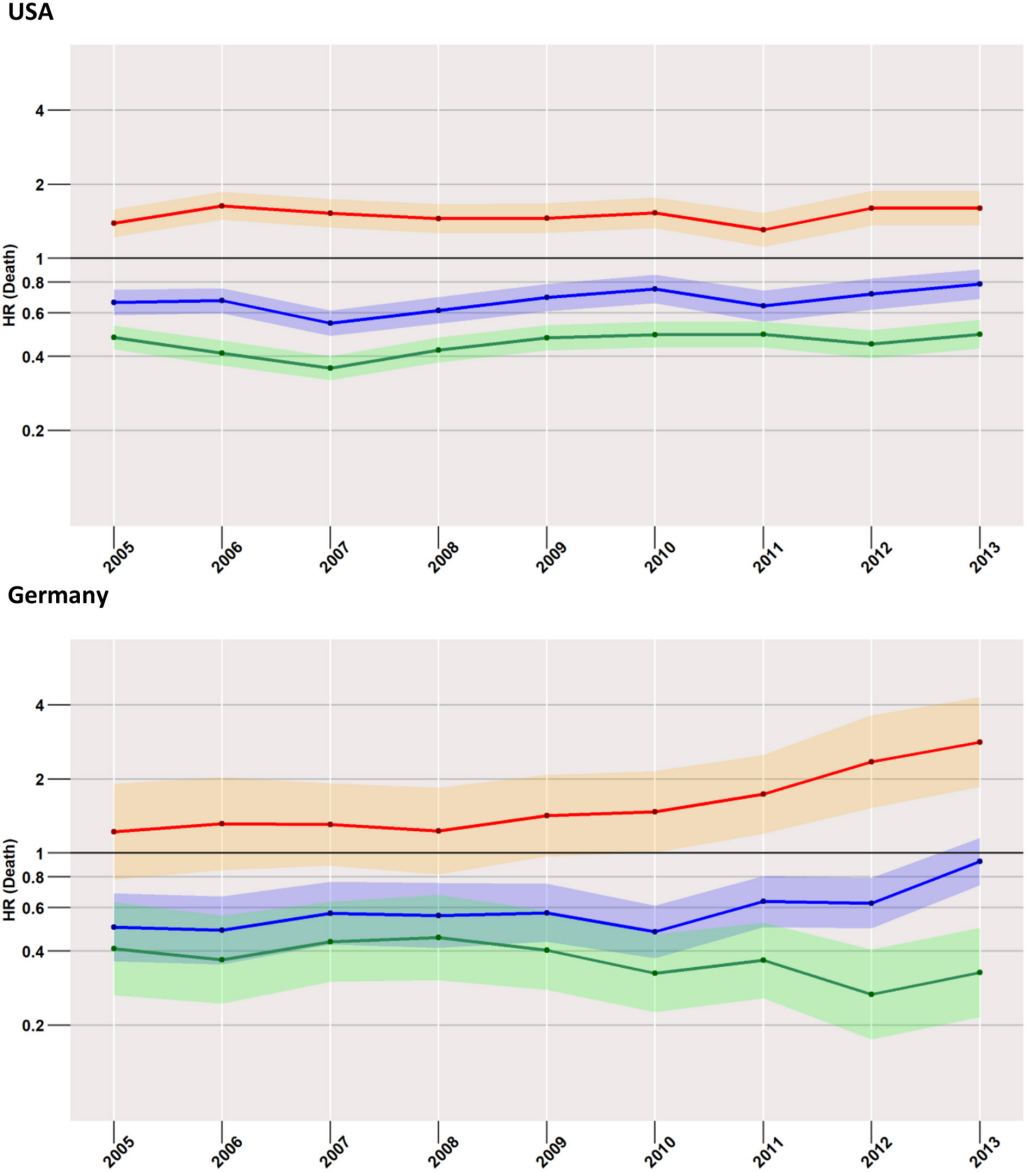
Treatment comparisons between radiotherapy, surgery, and no treatment for overall early mortality (24-months) of the years between 2005 and 2013 after the exclusion of cases with death from bladder cancer. Adjusted models, Red, surgery vs. radiotherapy; Blue, surgery vs. no treatment; Green, radiotherapy vs. no treatment.

After excluding cases that died from bladder cancer, we found little changes in the effect estimates (Figure 5). In the bias analysis of missing data, we found the pattern of missing data to differ considerably between SEER and German data. Most importantly to our results, treatment was only a minor predictor of missing data (Figure S1). Furthermore, estimates of piecewise regression analyses comparing surgery with radiotherapy were weaker in unadjusted models, indicating a bias against radiotherapy due to confounders such as older age in patients with radiotherapy (Figures S2A,B).

Finally, when we considered cases with low-risk prostate cancer and an age below 60-years in the US cohort, effect sizes of HRs and RDs decreased considerably (Figure S3, maximum HR: 4.6, 95% CI: 1.4–14.9; maximum RD: 0.9, 95% CI: 0.2–1.5).

Differences in estimates between imputed and complete data were only minimal (Figure S4), amounting to an average of 11.8% in the German data and 14.6% in the US data in year-specific 24-month early mortality analyses.

## Discussion

In summary, we found a considerably higher risk of early mortality in patients receiving surgery when compared to radiotherapy in both cohorts with very similar effect sizes. This effect was highest immediately after diagnosis and decreases slowly, but reaches a constant level not earlier than 24-months after diagnosis. The adverse effect related to surgery seems to extend far longer than suggested by the analysis performed in the previous studies discussed below. This finding was robust in subgroup analyses of low-risk patients.

Even more striking is the adverse survival prospect in the surgery group when the 3-month mortality is concerned. This effect is averaged out in later months by an equalizing and eventually inversing survival prospect in both groups resulting in an HR close to 1 in the long-term survival.

Regarding 30-day mortality following primary prostatectomy, Alibhai et al. analyzed the data of 11,010 men from the Ontario Cancer Registry (13). While total mortality was 0.48% for the whole cohort, absolute excess mortality was linked to patient's age, with 0.18% in the group aged 50–59-years vs. 0.59% for the group aged 70–79-years. The relative mortality risk was estimated to be 9-fold higher than the baseline risk in all age groups. These results confirm the previous work by Lu-Yao et al. that calculated a 30-day mortality of 1.04% for the age group of 75–79-years (14) In general, cardiovascular disease is supposed to be the major cause of 30-day mortality. In the abovementioned cohort of the Ontario region, 38% of deaths could be assigned to it (15).

In contrast, Alibhai et al. also assessed 30-day mortality after radiotherapy in 7,661 men. Interestingly, both mortality (0.1% for the whole cohort) and complication rates were lower for men receiving radiation therapy (16). Though age and number of comorbidities were more unfavorable in this group (compared to a cohort that underwent primary prostatectomy), this effect was consistent even without adjusting for these two confounders.

Hansen et al. compared the 30-, 60-, and 90-day mortality in 59,010 men after primary RP vs. RT when analyzing the SEER database for patients treated between 1998 and 2005 (17). The mortality risks were 5.2 (after 30-days, *p* < 0.001), 1.8 (after 60-days, *p* < 0.001), and 1.3 (after 90-days, *p* = 0.04) times higher after RP. The difference was especially pronounced in men >75-years and with a Charlson comorbidity index ≥2, with an increase of up to 3.2% in favor of radiotherapy.

It is important to stress that the early mortality differences mentioned above are related to cohorts with mixed risk groups regarding the staging of prostate cancer. The majority of patients are diagnosed with low-risk disease, which demonstrates excellent prognosis (18). Our results underline that a survival gap is also apparent in this group when surgery is compared to radiotherapy.

The best evidence from randomized trials concerning low-risk disease stems from the previously mentioned ProtecT study (6). Here, no apparent difference regarding all-cause mortality was found. Unfortunately, no survival plot is provided that would allow a crude estimation of early mortality. Based on our findings, we have to challenge a considerable efficacy gap between findings in standardized studies and the real-life experience as indicated by the registry data used. A recent study found a more favorable survival in patients with radical prostatectomy compared to Watchful Waiting with an HR of 0.55 (95% CI: 0.41–0.74) when long-term survival was addressed (19). Another study found an increase in restricted mean lifetime with prostatectomy compared to Watchful Waiting over follow-up time based on data of the SPCG-4 randomized trial, which is in part in line with our findings (20).

Coming to the comparison of no treatment to radiotherapy, we found a lower early mortality risk for patients receiving the latter in both the entire population and the low-risk subgroup. Patients in the no-treatment arm consist of two distinct groups, those that are eligible for active surveillance because of a low risk prostate cancer and those unfit for treatment because of a poor health condition.

It was only until 2010 when the SEER program started to collect and quality assure data regarding active surveillance, and its utilization nearly doubled from about 3.7% (2010) to 7.3% (2015) in the US (21).

The increasing use of active surveillance as treatment might be the reason for the decreasing survival gap in the US population for cases with no treatment in comparison to radiotherapy starting around 2009, when health conditions might have improved in the group.

On the other hand, we found a slightly better early mortality in the no-treatment group when we considered low-risk cases compared to surgery in the US population. In contrast, the mortality risk was higher in the no-treatment group in relation to surgery when we considered all stages. This might be because if patients in an advanced cancer stage fail to receive active treatment, they are predominately in a poor health condition.

In summary, the low-risk subgroup incorporates a relevant proportion of patients that are candidates for surveillance due to a favorable cancer stage compared to all cases. Here, the influence of therapy-limiting comorbidities on the decision for no treatment is substantial. This leads to a more pronounced influence of the periprocedural risk of surgery on early mortality in the low-risk group.

In general, the results of our analyses underline the importance of an individualized therapy decision for each patient with newly diagnosed prostate cancer in order to assure an effective and safe disease management.

### Bias and Limitations

Most importantly, when one compares treatments, the problem of unobserved confounders appears, which might easily bias results in favor of the treatment requiring a better health condition—in the present case, this is the surgical treatment. Still, as we observed a disadvantageous early mortality in subjects having received surgery, we would expect the results to be underestimated. Considering the recently published results of the ProtecT trial, (6) where no difference in the survival prospect was established, we might regard the HR beyond 36-months as the time when a steady state was reached that reflects the unaccounted bias. Thus, we would even underestimate the adverse early mortality found in cases with prostatectomy.

Furthermore, the limited case numbers in the German registry data lead to considerable statistical uncertainty, as is represented by wide CIs. However, estimates between both cohorts are comparable.

Respecting these limitations, we found a markedly higher short- to mid-term mortality in the group receiving surgery when compared to subjects having been treated by means of radiotherapy. These results stress a possible efficacy gap in terms of early mortality when results from clinical trials are related to real-life situations. Furthermore, surgical treatment should be restricted to patients that are in excellent clinical condition, especially in light of an adverse survival rate possibly not existing until 36-months after diagnosis.

## Data Availability Statement

The datasets analyzed in this manuscript are not publicly available. Requests to access the datasets should be directed to the Robert-Koch Institute, subject to data privacy.

## Author Contributions

DM performed the analyses and wrote the manuscript. DV wrote parts of the manuscript and read the manuscript critically. CD wrote parts of the manuscript.

### Conflict of Interest

The authors declare that the research was conducted in the absence of any commercial or financial relationships that could be construed as a potential conflict of interest.
