# Corrigendum: Early Mortality of Prostatectomy vs. Radiotherapy as a Primary Treatment for Prostate Cancer: A Population-Based Study From the United States and East Germany

**DOI:** 10.3389/fonc.2021.635433

**Published:** 2021-02-05

**Authors:** Daniel Medenwald, Dirk Vordermark, Christian T. Dietzel

**Affiliations:** Department of Radiation Oncology, University Hospital Halle (Saale), Halle (Saale), Germany

**Keywords:** early mortality, prostate cancer, prostatectomy, radiotherapy, general population

In the original article, there was a mistake in **Figure 1** as published. The color shade of the Kaplan-Meier survival curves was confused between the group with surgery and radiotherapy for the US data. The corrected **Figure 1** appears below.

**Figure 1:**
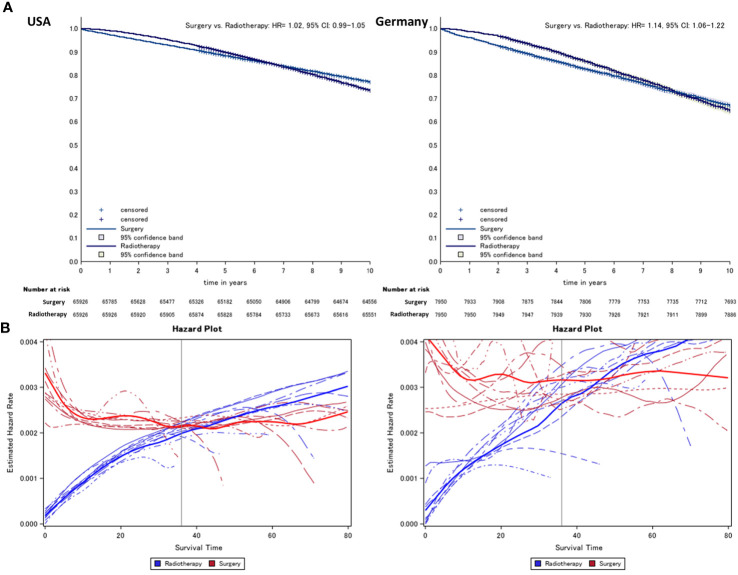
Kaplan–Meier **(A)** comparing surgery vs. radiotherapy (radiotherapy = reference) and **(B)** hazard survival plot of prostate cancer patients from German and US cancer registries. Lines indicate the year of diagnosis specific (2005–2013) conditional hazard for radiotherapy (risk to die in the following month, blue) and surgery (red) from Kaplan–Meier plots. Thick lines refer to the smoothed average of year-specific hazard in patients treated with radiotherapy and surgery, respectively.

The authors apologize for this error and state that this does not change the scientific conclusions of the article in any way. The original article has been updated.

