# Supplementary material for: Early Mortality of Prostatectomy vs. Radiotherapy as a Primary Treatment for Prostate Cancer: A Population-Based Study From the United States and East Germany
Source: Front Oncol. 2020 Jan 17;9:1451. doi: 10.3389/fonc.2019.01451 (PMC6978671; doi:10.3389/fonc.2019.01451)
Supplement: Supplementary file 1 [file Data_Sheet_1.pdf]

# *Supplementary Material*

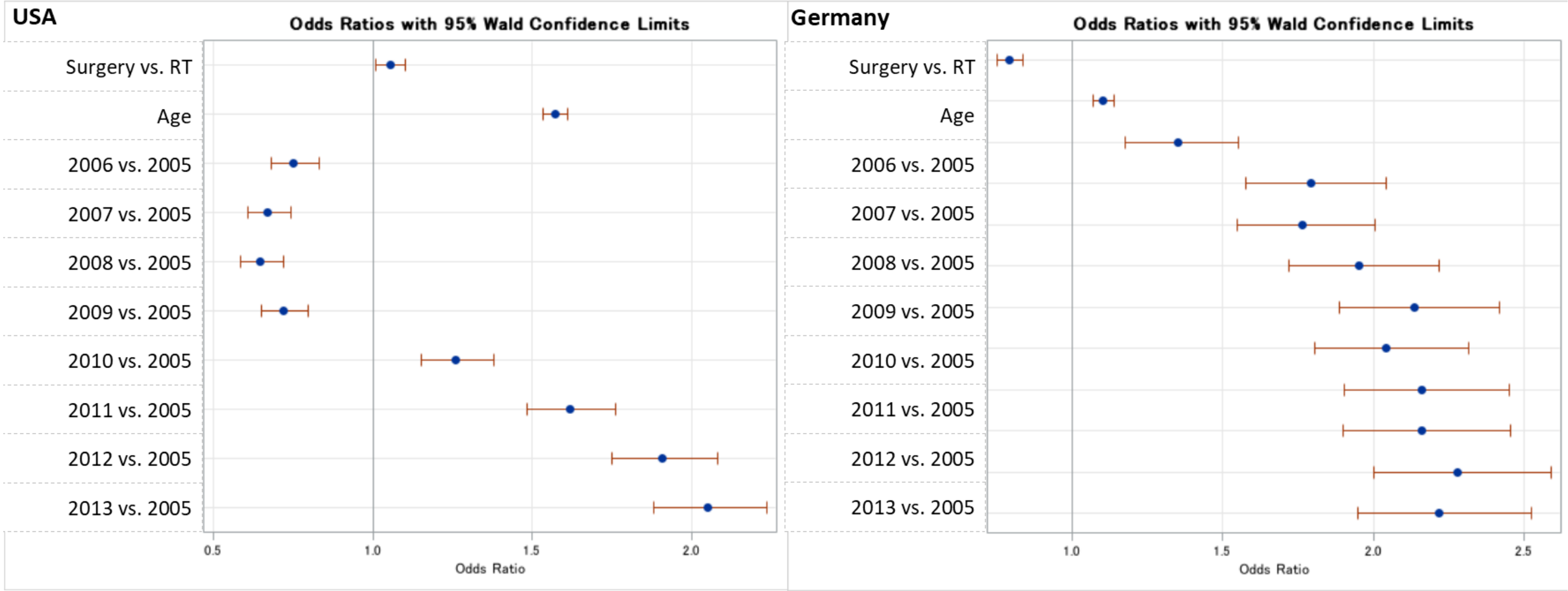


**Figure S1**: Pattern of missing data from multivariate logistic regression models predicting missing values.

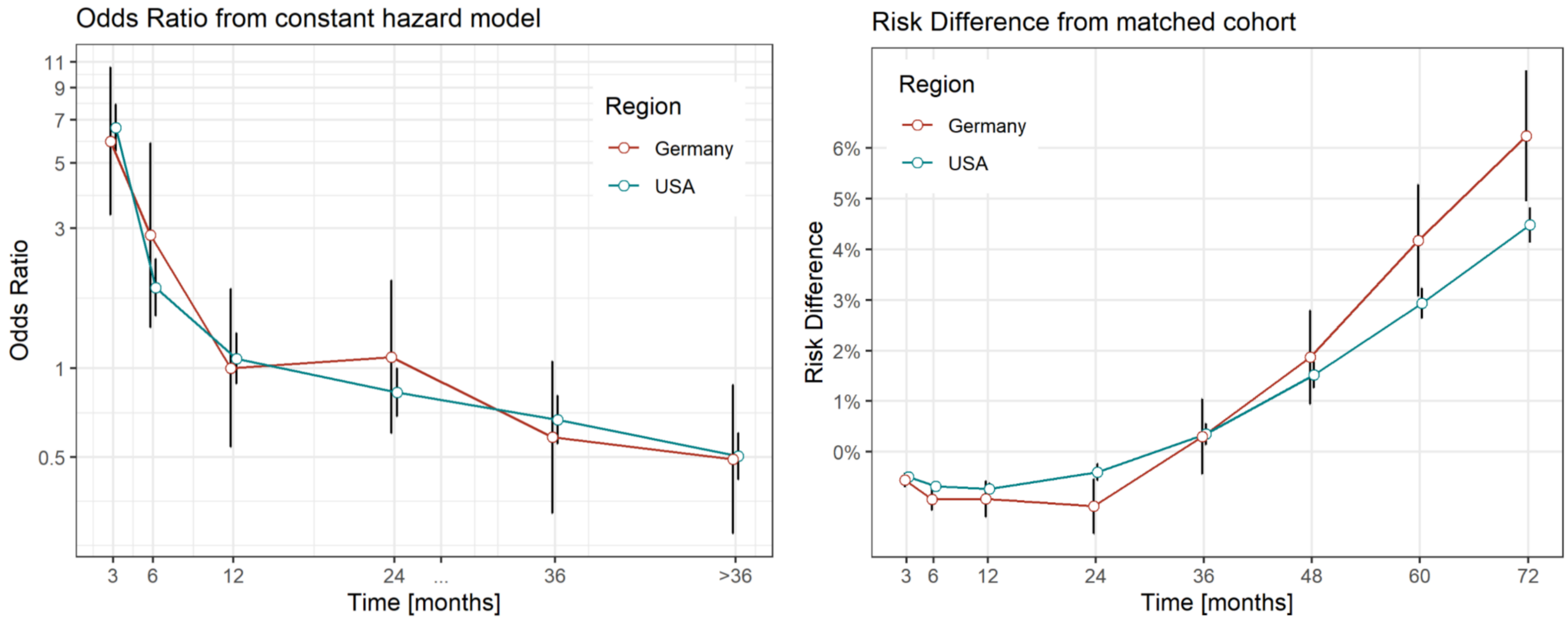


**Number of deaths/1000 person-years**

| Months | 3 | 6 | 12 | 24 | 36 |
|---|---|---|---|---|---|
| Radioth.[1] | 29.1 | 33.7 | 46.2 | 62.8 | 63 |
| Surgery[1] | 192.8 | 109.9 | 81.4 | 72.3 | 59.1 |
| HR[1] | 6.6 (5.5-7.9) | 1.9 (1.5-2.4) | 1.1 (0.9-1.3) | 0.8 (0.7-1) | 0.7 (0.6-0.8) |
| Radioth.[2] | 40 | 37.8 | 58.2 | 70 | 77.9 |
| Surgery[2] | 224.6 | 140.3 | 104.7 | 95.6 | 78.4 |
| HR[2] | 5.9 (3.3-10.5) | 2.8 (1.4-5.8) | 1 (0.5-1.9) | 1.1 (0.6-2.0) | 0.6 (0.3-1.1) |

| Months | 3 | 6 | 12 | 24 | 36 | 48 | 60 | 72 |
|---|---|---|---|---|---|---|---|---|
| RD[1] | -0.5 (-0.5--0.4) | -0.7 (-0.8--0.6) | -0.7 (-0.8--0.6) | -0.4 (-0.6--0.2) | 0.4 (0.1-0.6) | 1.5 (1.3-1.8) | 2.9 (2.6-3.2) | 4.5 (4.1-4.8) |
| RD[2] | -0.6 (-0.7--0.4) | -0.9 (-1.1--0.7) | -0.9 (-1.3--0.6) | -1.1 (-1.6--0.5) | 0.3 (-0.4-1) | 1.9 (0.9-2.8) | 4.2 (3.1-5.3) | 6.2 (4.9-7.5) |

**Figure S2:** Results of univariate unadjusted analyses. A) Results from piecewise regression models and crude risk differences from Kaplan-Meier estimates. B) Kaplan-Meier plots with hazard ratios computed from Cox regression models.

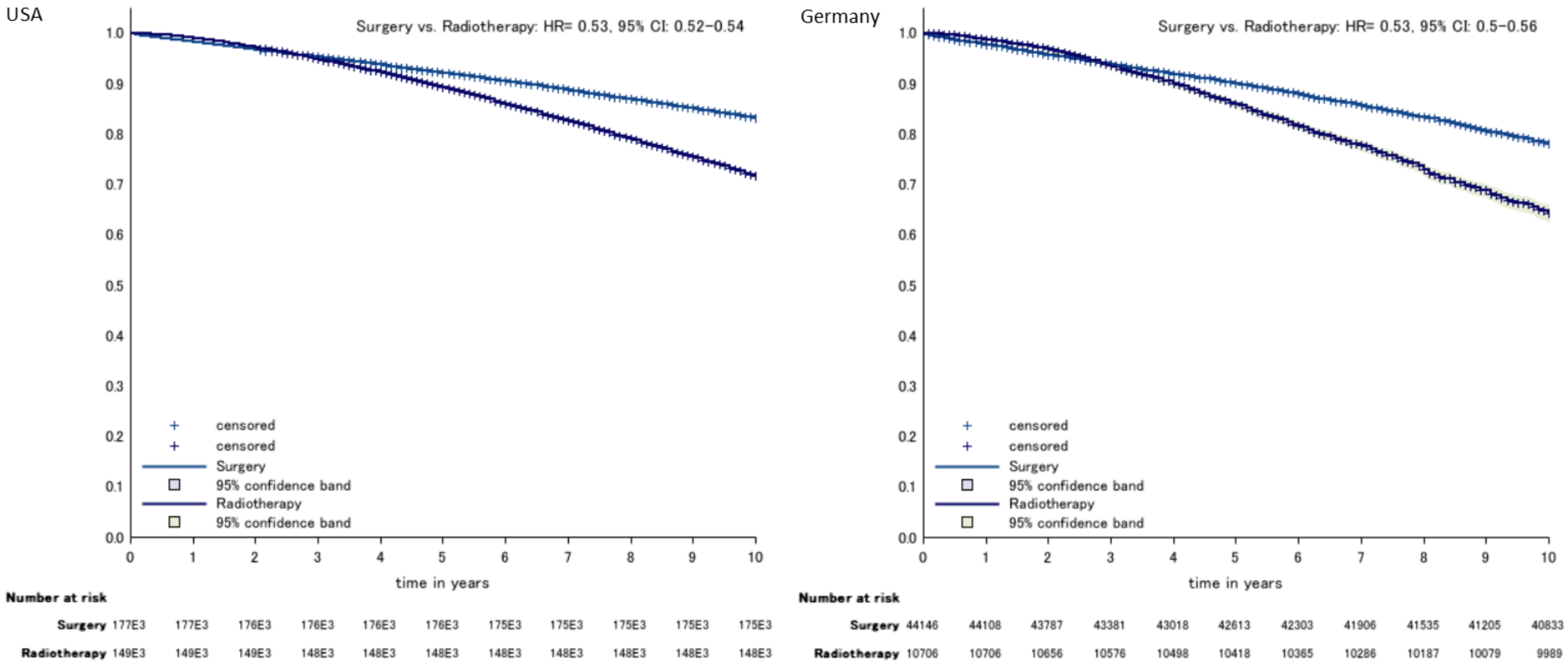


**Figure S3:** Sensitivity analysis of cases with low risk prostate cancer and an age below 60 at time of diagnosis.

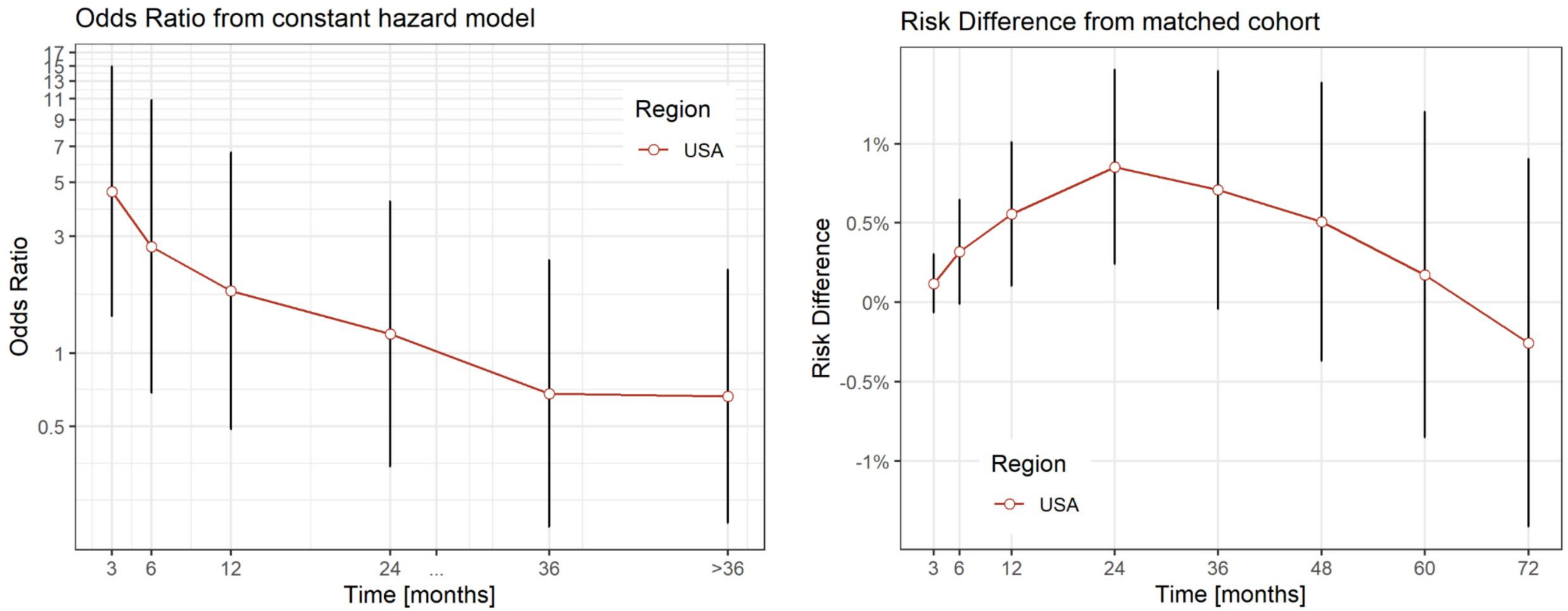


**Number of deaths/1000 person-years**

| Months | 3 | 6 | 12 | 20 | 36 |
|---|---|---|---|---|---|
| Radioth. | 29.1 | 33.7 | 46.2 | 62.8 | 63 |
| Surgery | 192.8 | 109.9 | 81.4 | 72.3 | 59.1 |
| HR | 4.6 (1.4-14.9) | 2.7 (0.7-10.8) | 1.8 (0.5-6.6) | 1.2 (0.3-4.2) | 0.7 (0.2-2.4) |

| Months | 3 | 6 | 12 | 24 | 36 | 48 | 60 | 72 |
|---|---|---|---|---|---|---|---|---|
| RD | 0.1 (-0.1-0.3) | 0.3 (0-0.6) | 0.6 (0.1-1) | 0.9 (0.2-1.5) | 0.7 (0-1.5) | 0.5 (-0.4-1.4) | 0.2 (-0.9-1.2) | -0.3 (-1.4-0.9) |

**Figure S4:** Results from complete case models. A) Results from piecewise regression models B) Results from Cox regression models analyzing the temporal course

**Table S1:** Numbers of proportions of missing data.

| | SEER data | Germany |
|---|---|---|
| Year of diagnosis | 0 | 0 |
| Survival time | 0 | 0 |
| TNM Stage | 10164 (2.30%) | 514 (0.72%) |
| Age | 0 | 0 |
| Grade | 12261 (2.78%) | 12593 (17.73%) |
| Treatment | 9892 (2.24%) | 0 |

**Table S2:** Basic characteristics after propensity score matching (complete data), * if selected by year-specific model

| **USA** | Radiotherapy | | Surgery | |
|---|---|---|---|---|
| | Mean [95% CI] | | Mean [95% CI] | |
| Age | 66.5 [66.5-66.6] | | 66.9 [66.8-70.0] | |
| Propensity Score | 0.564 [0.562-0.566] | | 0.564 [0.562-0.566] | |
| **Frequency values** | | | | |
| | n | % | n | % |
| Gleason ≤6 | 32399 | 50.5 | 31109 | 48.5 |
| Gleason >6 | 31747 | 49.5 | 33037 | 51.5 |
| | | | | |
| Locally limited (T1/T2) | 60249 | 93.9 | 59592 | 92.9 |
| Locally advanced (T3/T4) | 2907 | 4.5 | 3477 | 5.4 |
| Node-positive | 990 | 1.5 | 1077 | 1.7 |
| **Germany** | Radiotherapy | | Surgery | |
| | Mean [95% CI] | | Mean [95% CI] | |
| Age | 71.1 [71.0-71.3] | | 71.0 [70.9-71.2] | |
| Propensity Score | 0.350 [0.346-0.353] | | 0.351 [0.347-0.355] | |
| **Frequency values** | | | | |
| | n | % | n | % |
| Gleason ≤6 | 1194 | 75.4 | 1187 | 75.0 |
| Gleason >6 | 389 | 25.2 | 395 | 26.1 |
| | | | | |
| *Locally limited (T1/T2)* | 6906 | 86.9 | 6887 | 86.6 |
| *Locally advanced (T3/T4)* | 822 | 10.3 | 842 | 10.6 |
| *Node-positive* | 222 | 2.8 | 221 | 2.8 |
