# Supplementary material for: Early Mortality of Prostatectomy vs. Radiotherapy as a Primary Treatment for Prostate Cancer: A Population-Based Study From the United States and East Germany
Source: Front Oncol. 2020 Jan 17;9:1451. doi: 10.3389/fonc.2019.01451 (PMC6978671; doi:10.3389/fonc.2019.01451)
Supplement: Supplementary file 2 [file Table_1.DOCX]

|  | SEER data | Germany |
| --- | --- | --- |
| Year of diagnosis | 0 | 0 |
| Survival time | 0 | 0 |
| TNM Stage | 10164 (2.30%) | 514 (0.72%) |
| Age | 0 | 0 |
| Grade | 12261 (2.78%) | 12593 (17.73%) |
| Treatment | 9892 (2.24%) | 0 |
