# Supplementary material for: Early Mortality of Prostatectomy vs. Radiotherapy as a Primary Treatment for Prostate Cancer: A Population-Based Study From the United States and East Germany
Source: Front Oncol. 2020 Jan 17;9:1451. doi: 10.3389/fonc.2019.01451 (PMC6978671; doi:10.3389/fonc.2019.01451)
Supplement: Supplementary file 3 [file Table_2.docx]

| **USA*** | Radiotherapy | | Surgery | |
| --- | --- | --- | --- | --- |
|  | Mean [95% CI] | | Mean [95% CI] | |
| Age | 66.5 [66.5-66.6] | | 66.9 [66.8-70.0] | |
| Propensity Score | 0.564 [0.562-0.566] | | 0.564 [0.562-0.566] | |
| **Frequency values** |  |  |  |  |
|  | n | % | n | % |
| Gleason ≤6 | 32399 | 50.5 | 31109 | 48.5 |
| Gleason >6 | 31747 | 49.5 | 33037 | 51.5 |
|  |  |  |  |  |
| Locally limited (T1/T2) | 60249 | 93.9 | 59592 | 92.9 |
| Locally advanced (T3/T4) | 2907 | 4.5 | 3477 | 5.4 |
| Node-positive | 990 | 1.5 | 1077 | 1.7 |
| **Germany*** | Radiotherapy | | Surgery | |
|  | Mean [95% CI] | | Mean [95% CI] | |
| Age | 71.1 [71.0-71.3] | | 71.0 [70.9-71.2] | |
| Propensity Score | 0.350 [0.346-0.353] | | 0.351 [0.347-0.355] | |
| **Frequency values** |  |  |  |  |
|  | n | % | n | % |
| Gleason ≤6 | 5217 | 75.2 | 4990 | 73.8 |
| Gleason >6 | 1718 | 24.8 | 1773 | 26.2 |
|  |  |  |  |  |
| *Locally limited (T1/T2)* | 6906 | 86.9 | 6887 | 86.6 |
| *Locally advanced (T3/T4)* | 822 | 10.3 | 842 | 10.6 |
| *Node-positive* | 222 | 2.8 | 221 | 2.8 |

*complete cases only
